# Therapeutic Effect of Curcumol on Chronic Atrophic Gastritis (CAG) and Gastric Cancer Is Achieved by Downregulating SDF-1*α*/CXCR4/VEGF Expression

**DOI:** 10.1155/2022/3919053

**Published:** 2022-09-12

**Authors:** Xuehui Ma, Zhengbo Zhang, Xiayu Qin, Lingjing Kong, Wen Zhu, Lingzhi Xu, Xin Zhou

**Affiliations:** ^1^Department of Preclinical, Wuxi Hospital of Traditional Chinese Medicine, Wuxi, Jiangsu, China; ^2^Department of Gastroenterology, Wuxi Hospital of Traditional Chinese Medicine, Wuxi, Jiangsu, China; ^3^Health Examination Center, Wuxi Hospital of Traditional Chinese Medicine, Wuxi, Jiangsu, China

## Abstract

CAG is an essential procession of the transformation from gastritis into gastric cancer. A series of timely moves of diagnosis, treatment, and monitoring towards CAG to anticipate the potential population at risk of gastric cancer is an effective means to prevent gastric cancer occurrence. The main active monomer in Fuzheng Huowei Decoction is Curcumol, which is an indispensable ingredient in the treatment to CAG and gastric cancer. In this study, the CAG model, *in vitro* cultured gastric cancer cells, and participating nude mice were treated with Curcumol, and alterations in SDF-1*α*/CXCR4/VEGF expression were estimated using the assays of immunohistochemistry and Western blot. MTT, flow cytometry, transwell, HE staining, and tumor volume determination were applied for the verification of the regulatory effects of Curcumol on CAG and gastric cancer cells. The results showed that the expressions of VEGF, SDF-1*α*, CXCR4, and CD34 decreased in our CAG model with Curcumol treatment. Curcumol is in procession of an inhibitory effect toward the activity, migration, and invasion of gastric cancer cells, and it would also result in gastric cancer cells' apoptosis. We subsequently added SDF-1*α* overexpressing lentivirus to the Curcumol-treated group and found that the expressions of SDF-1*α*, CXCR4, and VEGF protein increased, and the inhibitory effect of Curcumol on gastric cancer cells was withdrawn. Our nude mouse experiment showed that Curcumol + SDF-1*α* group ended up with the largest tumor volume, while Fuzheng Huowei + NC group was with the smallest tumor volume. In conclusion, Curcumol is able to effectively protect the gastric tissue and suppress gastric cancer cells' viability. Curcumol functions as a therapeutic factor in chronic atrophic gastritis and gastric cancer by downregulating SDF-1*α*/CXCR4/VEGF expression.

## 1. Introduction

Gastric cancer is a malignant tumor caused by gastric epithelial lesions, with its second largest cancerous prevalence in the world. China is a country of high gastric cancer occurrence. The main task of gastric cancer research is to find and develop effective remedies. The occurrence of gastric cancer is a gradual process, which begins with the transformation of the normal gastric mucosa into gastric mucosa epithelial abnormalities or diseases. It is known as pregastric cancer, and eventually, it ends up as gastric cancer [1, 2]. CAG is a stage of precancerous disorder, characterized by the abnormal differentiation and proliferation of the gastric intrinsic gland or the intestinal metaplasia in the process of decay and proliferation. CAG is a process in which the gastric epithelial cells suffer repeated damage. It is caused by the long-term action of pathogenic factors on gastric mucosa, resulting in the reduction of inherent glands in gastric mucosa, accompanying with or without intestinal metaplasia and/or pseudopyloric metaplasia [3–5]. CAG is the junction amid chronic superficial gastritis and gastric cancer and is a key stage of gastric mucosa malignant transformation. Any timely attempt of the intervention of CAG stage is the key to reverse the occurrence of gastric cancer.

In view of the fact that there is currently no specific treatment for CAG clinically, this study aims to explore safe and effective drugs for CAG. The theory of traditional Chinese medicine points out that most patients with chronic atrophic gastritis have a long course of disease, which will ultimately lead to a certain degree of discord between chi and blood, causing blood stasis. Contextually, spleen deficiency with collateral stasis, decreasing gastric mucus secretion, and mucous membrane loss is gradually leading to gastric atrophy [6–8]. A moderate amount of the traditional Chinese medicine in CAG prescription is able to accelerate blood circulation and remove blood stasis, which effectively restores gastric mucous secretion and eliminates gastric atrophy.

Zedoary turmeric is a warm-property traditional Chinese medicine, tasting spicy and bitter, endowed with a strong affinity to the liver and spleen, holding the efficiency of dredging chi to break congestion, eliminating accumulation, and relieving pain. Modern phytochemical studies show that the main chemical constituents of Zedoary are volatile oil, curcumin, polysaccharides, sterols, phenolic acids, and alkaloids [9]. Of all these components, Curcumol is the main player with the highest content in the volatile oil of curcuma zedoaria. Curcumol has potential anticancer and anti-inflammatory properties, and it is involved in the regulation of angiogenesis and analgesia, promoting wound healing and antioxidant ability. Pharmacologically speaking, Curcumol has low toxicity to human body, and its high efficiency and low toxicity in the treatment of tumor is impressive [10, 11]. Curcumol is able to induce tumor cell apoptosis through different signaling pathways and effectively inhibit gastric cancer cell lines' proliferative activity, which has also been verified by animal studies. Currently, the therapeutic effect of Curcumol on CAG, also known as the precancerous lesion of gastric cancer, remains vacant.

Studies have shown that Curcumol promotes wound healing in a manner of eliciting the expression of VEGF in hyperglycemia rats. Besides, Curcumol represents a therapeutic effect on liver fibrosis. According to the theory of traditional Chinese medicine, gastric collateral stasis is one of the most important pathological factors of CAG, which is closely related to pathological changes, such as gastric epithelium and glandular atrophy, fibromuscular hyperplasia, intestinal metaplasia, and atypical hyperplasia, and it is also a key step in the development and malignant transformation of CAG. This study is committed to explore the therapeutic effects of Curcumol in the cell experiments with CAG and gastric cancer *in vivo* and *in vitro*.

## 2. Materials and Methods

### 2.1. Establishment of the CAG Animal Model

SD rats were fed adaptively for one week. The chronic atrophic gastritis model of rats was established as follows: 20 mmol/L of deoxycholate sodium solution (pH 7 to 7.8) was freely consumed, and 60% ethanol (2 mL/mouse) was gavaged every 10 days. 30% ethanol and 10 mmol/L were given alternately every 7 days from the 31^st^ day. After 12 weeks, gastric tissues were collected for examination. Animal experiments were authorized by the Animal Ethics Committee of Wuxi Hospital of Traditional Chinese Medicine (SWJWDW2021042601).

After modeling, the rats were split up into three groups (treatment group, model group, and control group), with 6 rats in each group. The rats in the treatment group were given 20 mg/kg of intragastric administration every day, while those rats in the model group and control group were administrated with the tantamount amount of normal saline in consecutive 28 days.

Model + Curcumol/Fuzheng Huowei decoction + SDF-1*α* overexpression AAV group was given with the simultaneous administration of the formulae of Curcumol/Fuzheng Huowei decoction on the same day after modeling. SDF-1*α* overexpressed AAV treatment was followed by tail vein injection (model + Zedoary turmeric enol/Fuzheng Huowei decoction + AAV control group injection of control virus, others are the same). All rats were sacrificed after 4 weeks of virus injection and were sampled.

### 2.2. Nude Mouse Tumorigenicity Assay

Thirty nude mice were injected with SGC7901 cells to establish the transplanted tumor. The tumor size and body weight of all mice were evaluated twice a week. After the tumor grew to about 0.5 cm in length, all mice were split up into 5 groups, with 6 mice in each group. They were the model control group, Curcumol treatment group, Curcumol + SDF-1*α* overexpression AAV group, Fuzheng Huowei decoction treatment group, and Fuzheng Huowei decoction + SDF-1*α* overexpression AAV group. Curcumol and Fuzheng Huowei formulae were administrated in the way of gastric instillation, while AAV was given by injection. Tumor growth curve was drawn in accordance with the parameters of tumor volume. On day 28, the mice were sacrificed and weighed for tumor detection.

The establishment of the gastric cancer cells subcutaneous transplanted tumor nude mice model: gastric cancer cells at logarithmic growth stage were prepared into a cell suspension with a concentration of 1 × 10^7^/mL, and 170 *μ*L of the suspension was inoculated subcutaneously into the right axillary region of nude mice. When the length of the tumor reached about 0.5 cm, the mice were grouped. They were the model control group, Curcumol treatment group, Curcumol + SDF-1*α* overexpression AAV group, Fuzheng Huowei decoction treatment group, and Fuzheng Huowei decoction + SDF-1*α* overexpression AAV group. Moreover, Curcumol and Fuzheng Huowei decoction were administered in the way of gastric perfusion, while AAV was given by injection.

### 2.3. HE Staining

After fetching, the samples were rinsed with PBS and fixed with 4% paraformaldehyde. The HE staining kit was the product of BEYOTIME Company, and the test was carried out according to the kit instructions.

### 2.4. Immunohistochemical Analysis

Paraffin sample sections were dewaxed by xylene and gradient ethanol. The samples were then immersed in 0.01 mol/L citrate buffer (pH 6.0) for antigen repairing. The slices were then immersed in 3% hydrogen peroxide to block endogenous catalase and sealed with 5% goat serum in a wet box at room temperature. The samples were then added and incubated with primary antibody at 4°C overnight. After being washed by PBS for 3 times, the second antibody was added and incubated with it at 37°C for 1 h. The slices were then washed and restained with hematoxylin, dehydrated, and sealed.

### 2.5. MTT Detection on Cellular Viability

SGC7901 cells were provided by the Chinese Academy of Sciences, cultured with the complete medium containing 10% FBS and 1% penicillin-streptomycin. The cells were grown in 96-well plates with 200 *μ*L per well, and 3 separate wells were set for each group. After incubation for 6 h, 24 h, and 48 h, the 96-well plates were taken out of the incubator, and 20 *μ*L of 5 mg/mL MTT solution was added to each well. The culture plate was incubated in the incubator for 4 h, and then the culture was terminated. The culture medium was removed from the culture wells, and 150 *μ*L of dimethyl sulfoxide was added to each well, and the crystals were fully dissolved by shaking on a shaker at low speed for 10 min. The absorbance value of each well was measured at OD490 nm of ELISA.

(1) $$\begin{array}{c} \text{Cell viability}\left(\%\right)=\frac{\left[A\left(\text{experimental group}\right)-a\left(\text{blank group}\right)\right]}{\left[A\left(\text{control group}\right)-A\left(\text{blank group}\right)\right]}\times 100\%,\\ \text{Cell inhibition rate}=\frac{\left[A\left(\text{control group}\right)-a\left(\text{experimental group}\right)\right]}{A\left(\text{control group}\right)}=1-\text{cell viability.} \end{array}$$

### 2.6. Flow Cytometry Detection on Apoptosis

SGC7901 cells were divided as follows: control group, Curcumol treatment group 100 mg/L (48 h), and Fuzheng Huowei decoction treatment group 100 mg/L (48 h). The apoptosis of SGC7901 cells was evaluated via flow cytometry. After the cells were harvested, the cells were centrifuged at 300 g for 5 min, and the supernatant was discarded. The cells were then rinsed once with PBS, and the supernatant was discarded after centrifugation. The supernatant was suspended with 100 *μ*L of diluted 1 × Annexin V Binding Buffer. 2.5 *μ*L Annexin V-APC and 2.5 *μ*L DAPI staining solution were added to the cell suspension. The cells were incubated at room temperature and kept in dark for 15 min. 400 *μ*L of Annexin V Binding Buffer was added, and the samples were mixed and tested immediately.

### 2.7. Transwell Assay Detection on Cell Migration and Invasion

Cell migration experiment: 1 mL trypsin containing EDTA was added to digest SGC7901 cells. After the digestion was complete, culture medium containing serum was added to terminate digestion. The culture medium was removed by centrifugation, the cells were rinsed with PBS once or twice and suspended with serum-free culture to adjust the cell density to 5 × 10^5^/mL. 100 *μ*L cell suspension was added to each well and stimulated with drugs. 500 *μ*L complete medium containing 20% FBS was added to the lower chamber, and the cells were incubated at 37°C for 24 h. Transwell chambers were removed, the culture medium in the well was discarded, and the cells were washed twice with calcium-free PBS. The cells were fixed with 4% paraformaldehyde for 20 min. After washing with PBS, the cells were stained with 0.1% crystal violet for 20 min. Cells in the middle and surrounding 5 fields were counted under a 100-fold microscope, and their average values were taken.

Cell invasion assay: 300 *μ*L serum-free medium was taken, 60 *μ*L Matrigel was added to the medium and mixed, and 100 *μ*L was added to the upper chamber and incubated in a 37°C incubator for 5 h. 1 mL of trypsin, containing EDTA, was added into the cell wells to digest the SGC7901 cells. After digestion, the cell density was adjusted to 5 × 10^5^/mL. Matrigel was washed once in serum-free medium, 100 *μ*L cell suspension was added to each well, and 500 *μ*L of the complete medium containing 20% FBS was added into the lower chamber, which was incubated at 37°C for 24 h. The transwell chamber was taken out, and the culture medium in the well was removed. It was rinsed twice with calcium-free PBS, fixed with 4% paraformaldehyde for 20 min, stained with 0.1% crystal violet for 20 min after PBS cleaning, and the unmigrating cells in the upper layer of the chamber were gently wiped with wet cotton ball and cleaned with PBS for 3 times. Five fields of the cells in the middle and surrounding areas were counted under a 100-fold microscope and their average values were taken.

### 2.8. Western Blot Detection on Protein Expression

RIPA lysate was added to the sample, which was shaken on the vortex for 1 min and placed on ice for 10 min. It was then centrifuged at 13,000 rpm at 4°C for 20 min. 500 *μ*g of total protein from each sample was mixed with 5 × SDS loading buffer. The concentrated glue was run with 80 V, and then the voltage was converted to 120 V, until bromophenollan was just transferred to the bottom of the glue plate. The PVDF membrane was cleaned with TBST for 1 min and then sealed with 5% skim milk sealer at room temperature for 1 h. The primary antibody was diluted at 1 : 1000, with the primary antibody diluent and incubated with the sealed PVDF membrane at 4°C overnight. The secondary antibody was diluted into a certain concentration (1 : 2000) with the blocking solution, and then kept with the PVDF membrane at room temperature for 1 h. The ECL exposure solution was added on the whole membrane. After 1-min reaction with PVDF membrane, the ECL exposure solution was put into the exposure instrument for exposure detection.

## 3. Results

### 3.1. Curcumol Inhibited VEGF, SDF-1*α*, CXCR4, and CD34 Expressions in the CAG Model

**Figure 1:**
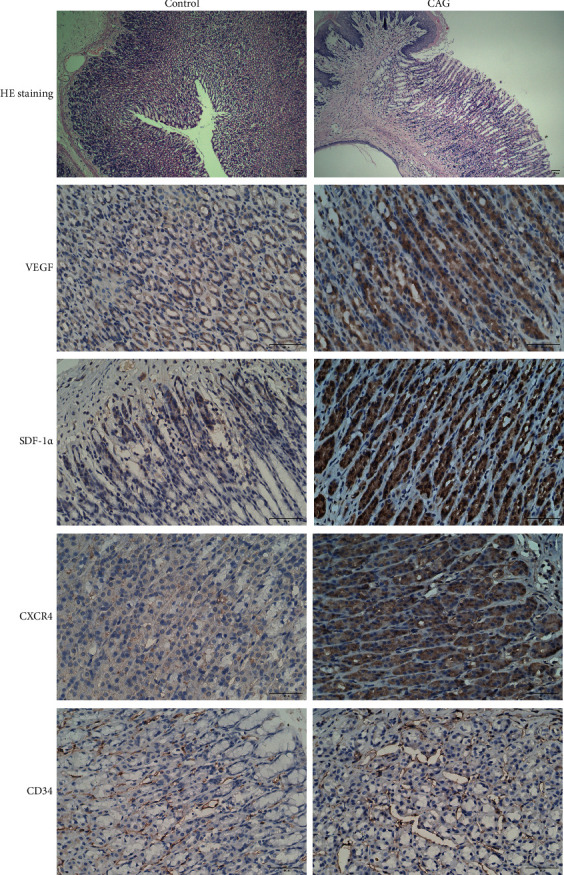
CAG model establishment and relevant detection. Tissue structures and marker protein expressions of CAG model animals were tested by HE staining and immunohistochemistry. *N* = 3, Scale bar: 50 *μ*m.

In this study, the animal model of CAG was firstly prepared using ethanol feeding, and then the tissue structure and marker protein expressions of model animals were detected via HE staining and immunohistochemistry. Then, we found that the gastric tissues of the animals in the CAG model group was disordered and irregular, and there was obvious inflammatory cell infiltration. Immunohistochemical assay revealed that the VEGF, SDF-1*α*, CXCR4, and CD34 expressions in the CAG model group were signally elevated versus control groups (Figure 1).

**Figure 2:**
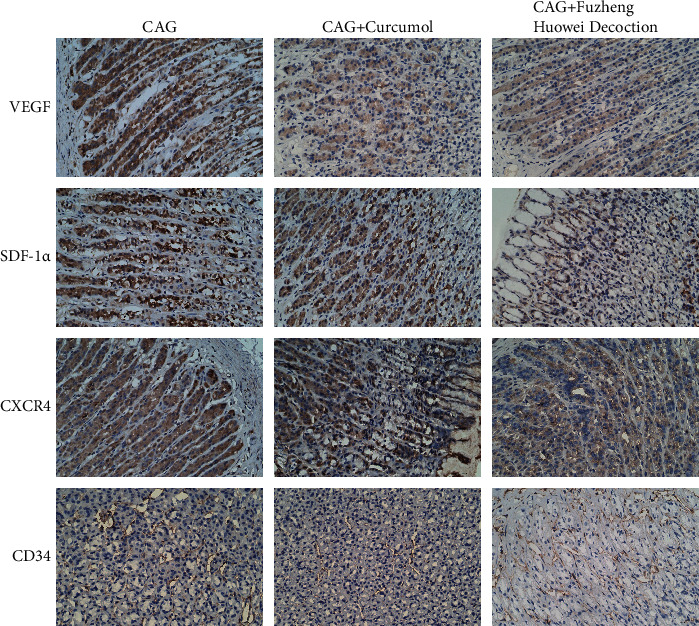
Regulations of Curcumol on relevant protein expressions in CAG model. The expression levels of VEGF, SDF-1*α*, CXCR4, and CD34 in CAG model were assessed by immunohistochemistry. *N* = 3, Scale bar: 50 *μ*m.

Subsequently, Curcumol and Fuzheng Huowei decoction were, respectively, administrated to the model group. Then, immunohistochemistry detection on the expressions of the related proteins was carried out. The results showed that when compared to the model group, either the VEGF, SDF-1*α*, CXCR4, and CD34 expressions in Curcumol or positive drug groups were decreased (Figure 2).

### 3.2. Curcumol Inhibits the Activity, Migration, and Invasion of Gastric Cancer Cells

**Figure 3:**
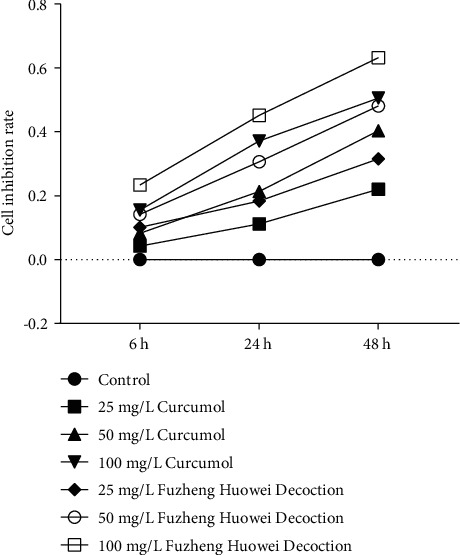
MTT determination on gastric cancer cell viability. *N* = 3.

In this section, we demonstrated curcumin's regulatory function in the gastric cancer cells cultured *in vitro*. Cells were given 25 mg/L, 50 mg/L, and 100 mg/L Curcumol/Fuzheng Huowei decoction. The results of MTT assay reflected that the inhibition rate of 100 mg/L Fuzheng Huowei decoction for 48 h was the highest, and the subsequent was 100 mg/L Curcumol for 48 h (Figure 3). According to this finding, we chose Curcumol treatment group 100 mg/L (48 h) and Fuzheng Huowei decoction treatment group 100 mg/L (48 h) to carry out our the experiments further.

**Figure 4:**
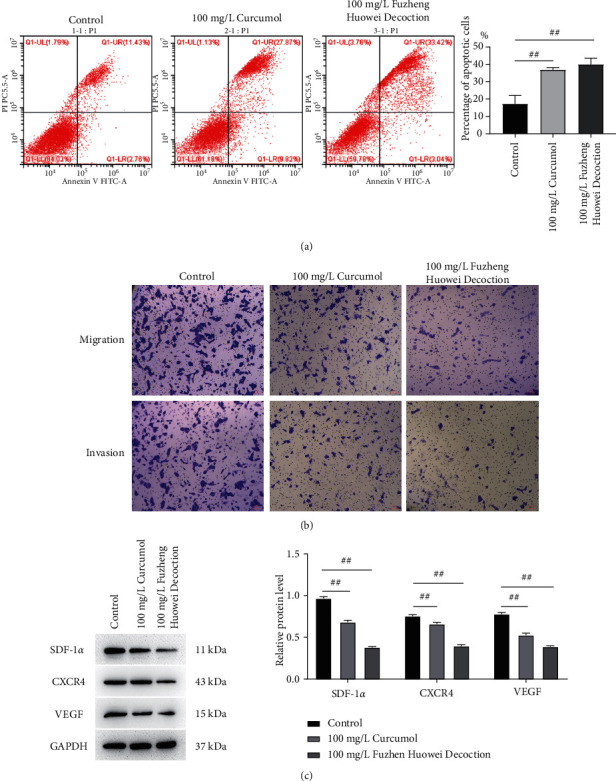
Inhibitory effects of Curcumol on apoptosis, migration, and invasion of gastric cancer cells. (a) Flow cytometry determination on cell apoptosis. (b) Determination of Transwell assay on gastric cancer cell migration and invasion. (c) Determination of Western blot on the protein expression levels of SDF-1*α*, CXCR4, and VEGF in gastric cancer cells. *N* = 3, Scale bar: 50 *μ*m.

The apoptosis rate of gastric cancer cells in the control group, Curcumol treatment group, and Fuzheng Huowei decoction treatment group were 17.32%, 36.92%, and 39.99% in several. Curcumol and Fuzheng Huowei decoction significantly induced the apoptosis of gastric cancer cells (Figure 4(a)). Gastric cancer cells' migratory and invasive abilities were evaluated via the transwell assay, and it turned out that the number of migratory and invasive cells in the cells treated with Curcumol and Fuzheng Huowei decoction decreased significantly (Figure 4(b)). In-depth detection on SDF-1*α*, CXCR4, and VEGF protein expressions showed that the SDF-1*α*, CXCR4, and VEGF protein levels in gastric cancer cells were greatly retarded after the treatment with Curcumol and positive drug Fuzheng Huowei decoction (Figure 4(c)).

### 3.3. SDF-1*α* Reverses the Inhibition from Curcumol on Gastric Cancer Cells

**Figure 5:**
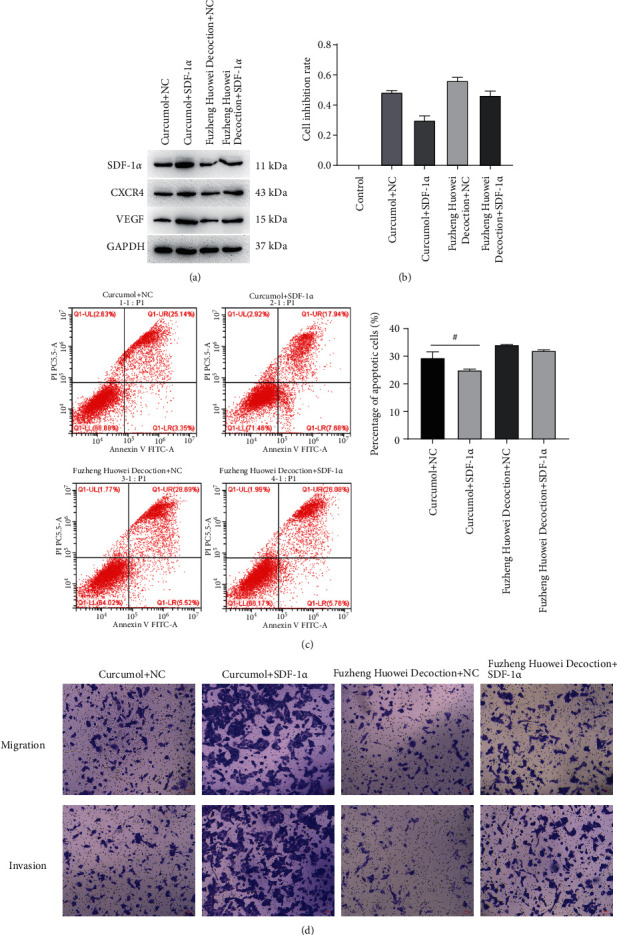
Reversed effect of SDF-1*α* toward Curcumol's inhibition on gastric cancer cells. (a) Western blot detection on the protein expression levels of SDF-1*α*, CXCR4, and VEGF. (b) MTT detection on cell viability. (c) Flow cytometry detection on cellular apoptosis rate. (d) Detection of gastric cancer cell migration and invasion by Transwell test. *N* = 3, Scale bar: 50 *μ*m.

In this section, we first treated gastric cancer cells with SDF-1*α* overexpressed lentivirus and Curcumol, and then Curcumol's regulatory effect on SDF-1*α*/CXCR4 axis was verified. Afterwards, our results indicated that high levels of SDF-1*α* elevated SDF-1*α*, CXCR4, and VEGF protein levels in gastric cancer cells (Figure 5(a)). The assay of MTT unveiled that the inhibition from Curcumol or Fuzheng Huowei decoction on the gastric cancer cells withdrew after the overexpression of SDF-1*α* (Figure 5(b)). Flow cytometry also revealed that when compared with Curcumol + empty virus group, the apoptosis rate of SDF-1*α* overexpressed + Curcumol group was significantly reduced (Figure 5(c)). Moreover, the number of migratory and invasive gastric cancer cells was signally increased (Figure 5(d)).

**Figure 6:**
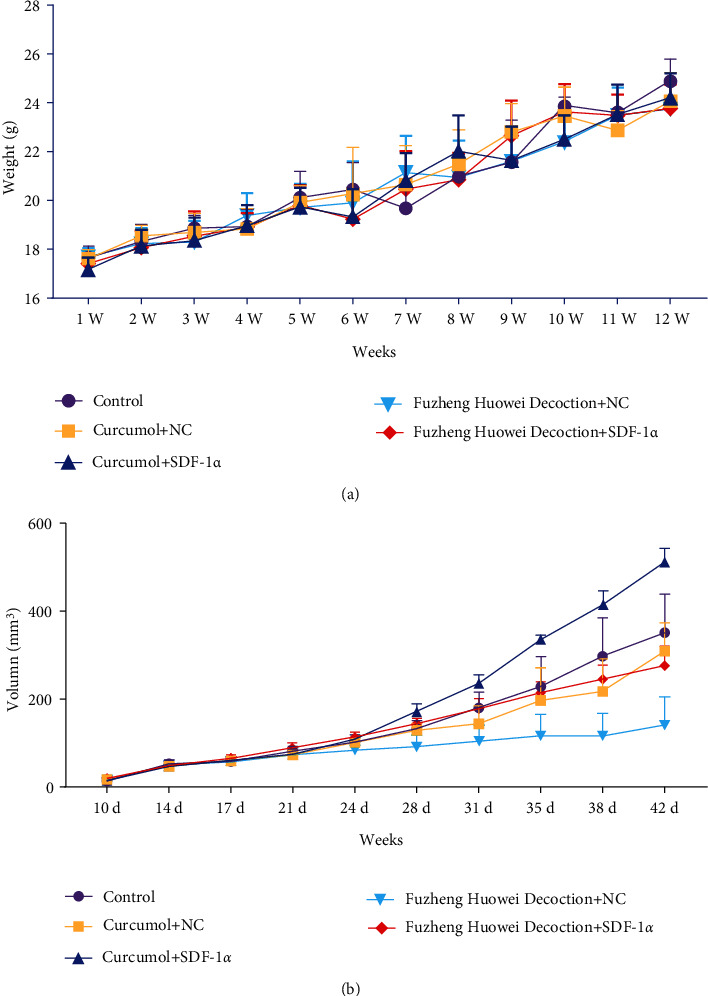
Results of body weight and tumor volume in tumorigenesis test on nude mice. (a) Weight measurement results of nude mice. (b) Tumor volume detection results of nude mice. *N* = 6.

**Figure 7:**
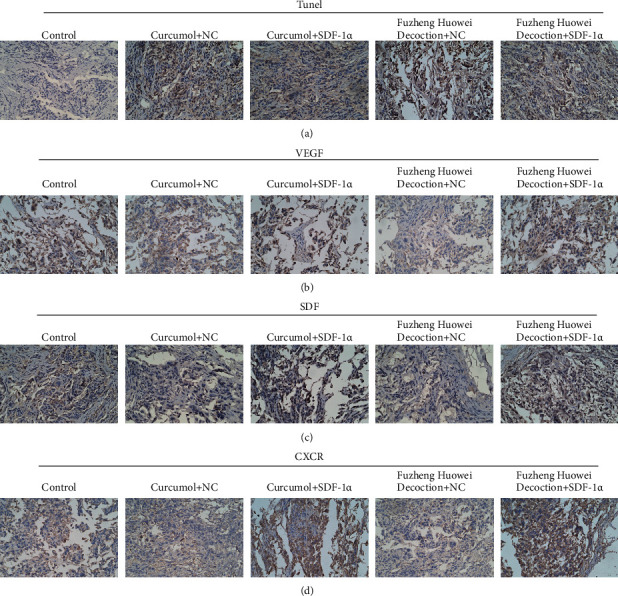
TUNEL and immunohistochemistry detection on apoptosis and protein expression of each group after treatment. *N* = 3, Scale bar: 50 *μ*m.

Finally, we further verified the regulation of SDF-1*α* on gastric cancer cells with the tumor-forming assay in nude mice. It turned out that there was no notable change in the weight of nude mice in the control group, Curcumol + NC group, Curcumol + SDF-1*α* group, Fuzheng Huowei decoction + NC group, and Fuzheng Huowei decoction + SDF-1*α* group during the test (Figure 6(a)). However, there were significant differences in tumor volume among all groups, which were that Curcumol + SDF-1*α* group had the largest tumor volume and Fuzheng Huowei decoction + NC group had the smallest tumor volume (Figure 6(b)). TUNEL and immunohistochemistry were applied for the detection onto the apoptosis and expression of marker proteins in each group after treatment. It was found that SDF-1*α*, CXCR4, and VEGF protein levels in the overexpressed SDF-1*α* group were raised, and the apoptosis rate was lower than that in the corresponding empty virus treatment (Figure 7).

## 4. Discussion

CAG is a necessary process from gastritis to gastric cancer. The diagnosis, treatment, and monitoring of CAG would effectively prevent the occurrence of gastric cancer [12, 13]. Compared with synthetic compounds, this traditional Chinese medicine component has the characteristics of less toxicity and more effects. In the preliminary clinical study, Fuzheng Huowei decoction has been proved to be effective in alleviating the clinical symptoms of CAG patients, boasting the effects of improving histological lesions and promoting mucosal repairing. Curcumol is an important monomer component of Fuzheng Huowei decoction, however, its regulatory mechanism on CAG and gastric cancer is still unknown. This study showed that Curcumol represses the VEGF, SDF-1*α*, and CXCR4 expressions in both CAG model and gastric cancer cells, and it also suppresses the activity, migration, and invasion of gastric cancer cells, inducing the apoptosis of gastric cancer cells.

Relevant studies have pointed out that the activation of SDF-1*α*/CXCR4 axis in either human breast cancer cell lines or prostate cancer cell lines promotes VEGF secretion to participate in tumor angiogenesis [14, 15]. In addition, it has been reported that peritoneal metastasis in gastric cancer proves to be related to the interaction between VEGF, CXCR4, and CXCL12. CXCR4 is a specific receptor of chemokine stromal cell derived factor (SDF-1*α*), which is involved in tumor growth, invasion, and metastasis. Angiogenesis mediated by SDF-1 binding to CXCR4 promotes tumor growth. It was found in the clinical sample studies of gastric cancer that positive SDF-1 and CXCR4 expression rates gradually increases along with gastric cancer progression, which was basically synchronous with the expression trend of VEGF in gastric cancer progression [16–18]. Our study shared a consistence with these findings above. We demonstrated that Curcumol would simultaneously reduce VEGF, SDF-1*α*, and CXCR4 protein levels in the CAG animal model, and the relevant outcomes from *in vivo* and *in vitro* experiments of gastric cancer were alike.

ATP-sensitive K+ channels couple intracellular metabolism with membrane excitability. These channels are inhibited by ATP. Hence, they open in low metabolic states and close in high metabolic states, resulting in membrane depolarization and triggering responses, such as insulin secretion, the modulation of vascular smooth muscle, and cardioprotection. The channel comprises four Kir6.x subunits and four regulatory sulphonylurea receptors (SUR). Different mechanisms have been proposed for the pathophysiology of gastric ulceration, including changes in the submucosal blood flow of the stomach, gastric motility, and acidity. It seems probable that adenosine 5′-triphosphate (ATP)-dependent potassium channels (K(ATP)) have a regulatory effect on the above factors [19]. Sulfonylurea receptor 1 (SUR1) is the regulatory subunit of ATP-sensitive potassium channels (KATP channels) and the receptor of antidiabetic drugs, such as glibenclamide, which induce insulin secretion in pancreatic *β* cells.

Targeting SUR1 with glibenclamide suppressed cell growth, cell-cycle progression, epithelial-mesenchymal transition (EMT), and cell migration. Moreover, SUR1 directly interacted with p70S6K and upregulated p70S6K phosphorylation and activity. In addition, glibenclamide inhibited p70S6K, and the overexpression of p70S6K partially reversed the growth-inhibiting effect of glibenclamide. Furthermore, glibenclamide upregulated the expression of the tumor suppressor Krüppel-like factor 4 (KLF4), and silencing KLF4 partially reversed the inhibitory effect of glibenclamide on cell growth, EMT, and migration [20].

Curcumol, a sesquiterpene isolated from Curcuma zedoaria, is known to possess a variety of health and medicinal values, which includes neuroprotection, anti-inflammatory, antitumor, and hepatoprotective activities. It inhibits NF-*κ*B activation by suppressing the nuclear translocation of the NF-*κ*B p65 subunit and blocking I*κ*B*α* phosphorylation and degradation. Taken together, the combination of *Curcuma zedoary* and kelp could inhibit the proliferation and metastasis of liver cancer cells *in vivo* and *in vitro* by inhibiting endogenous H2S production and downregulating the pSTAT3/BCL-2 and VEGF pathway, which provides strong evidence for the application of *Curcuma zedoary* and kelp in the treatments of liver cancer [21]. Curcumol inhibits the proliferative ability of SKOV3 cells, and the mechanism may be in association with the phosphatidylinositol 3-kinase (PI3K)/protein kinase B (Akt) pathway. Curcumol naturally suppresses the proliferation, migration, and invasion of SKOV3 cells and induces apoptosis. MTOR is a downstream factor beneath the PI3K/Akt pathway and has a regulatory effect on cell proliferation. In this study, we found that Curcumol significantly reduces the SDF-1*α*/CXCR4/VEGF protein levels in gastric cancer cells. Furthermore, the overexpression of SDF-1*α* brought about an upregulation in SDF-1*α*, CXCR4, and VEGF protein levels, and Curcumol's inhibitory effect on gastric cancer cells was withdrawn. In this study, we proved that Curcumol has therapeutic effects on CAG and that it reverses gastric cancer progression by manipulating the angiogenic activity mediated by the SDF-1/CXCR4 axis. It is the first study on the molecular mechanism of Curcumol regulating CAG and gastric cancer. It has laid a good research foundation for the clinical application of Curcumol. However, this study also has certain limitations. We have not been able to demonstrate the direct regulatory target of Curcumol and lack a more in-depth mechanism of exploration. This is our future research direction.

## 5. Conclusion

In this paper, we disclosed the fact that Curcumol functions as an inhibitor for gastric cancer cells by inhibiting gastric cancer cells' activity, migration, and invasion and inducing their apoptosis. Moreover, the expression levels of VEGF, SDF-1*α*, and CXCR4 decreased after Curcumol treatment. Our nude mouse experiment showed that Curcumol + SDF-1*α* group had the largest tumor volume, while Fuzheng Huowei + NC group ended up with the smallest. In conclusion, Curcumol effectively protects the gastric tissue and inhibits gastric cancer cell viability. Curcumol treats chronic atrophic gastritis and gastric cancer by decreasing SDF-1*α*/CXCR4/VEGF expression.

## Data Availability

The data used to support the findings of this study are included within the article.
